# Erythema Nodosum: An Unusual Clinical Manifestation of Oropharyngeal Tularemia

**DOI:** 10.7759/cureus.10602

**Published:** 2020-09-22

**Authors:** Armen Kishmiryan, Jeevan Gautam

**Affiliations:** 1 Infectious Disease, Nork Infectious Clinical Hospital, Yerevan, ARM; 2 Internal Medicine, Institute of Medicine, Kathmandu, NPL

**Keywords:** erythema nodosum, buboes, oropharyngeal tularemia, armenia, case report

## Abstract

Tularemia is a zoonotic infection caused by *Francisella tularensis*. Oropharyngeal tularemia, one of the several clinical forms of tularemia identified, is typically characterized by fever, sore throat, cervical buboes, and rarely, cutaneous lesions. Here we describe an uncommon clinical manifestation of oropharyngeal tularemia with erythema nodosum, an inflammatory condition that causes tender nodules to form on the lower legs. A 45-year-old woman with fever, sore throat, unilateral cervical buboes, and erythema nodosum on both legs was diagnosed with oropharyngeal tularemia based on clinical manifestations and positive latex agglutination testing. We prescribed a 14-day course of intramuscular streptomycin, which resulted in the complete recovery of the patient. It is unusual for tularemia to manifest with erythema nodosum as a primary symptom, particularly one that persists throughout the illness. Although the cause of erythema nodosum is unknown in nearly half of cases, it is important to identify or exclude possible infectious causes of this condition, including tuberculosis, Valley fever, cat scratch disease, and, as illustrated in the case described herein, tularemia.

## Introduction

Tularemia is a zoonotic bacterial disease caused by *Francisella tularensis*, a gram-negative coccobacillus, and transmitted by a tick or fly bite, exposure to infected animal tissue, or contact with infected mucous membranes [1]. The organism is transmitted by skin inoculation, inhalation, or ingestion, which also determines the type of tularemia a person develops. Human-to-human transmission is unlikely [2]. The disease can be potentially severe and life-threatening but is easily treatable with antibiotics [1].

Tularemia is prevalent in the northern hemisphere mostly, countries in eastern Europe, and continental western Europe [3]. The Republic of Armenia considers tularemia a public health priority, as the disease is endemic across >90% of the country. Since 2001, 262 cases of tularemia have been reported in Armenia: 182 (69%) cases of glandular form, 8 (3%) anginal-bubonic, and 75 (28%) oculoglandular form with farmers (49.6%), and rural population (93.6%) being the most affected ones [4]. Oropharyngeal tularemia is typically characterized by fever, sore throat, mouth ulcers, and cervical buboes, and rarely, cutaneous lesions and other skin conditions. Here we describe an uncommon clinical manifestation of oropharyngeal tularemia with erythema nodosum, an inflammatory condition that causes tender nodules to form on the lower legs and briefly review the relevant literature.

## Case presentation

**Figure 1:**
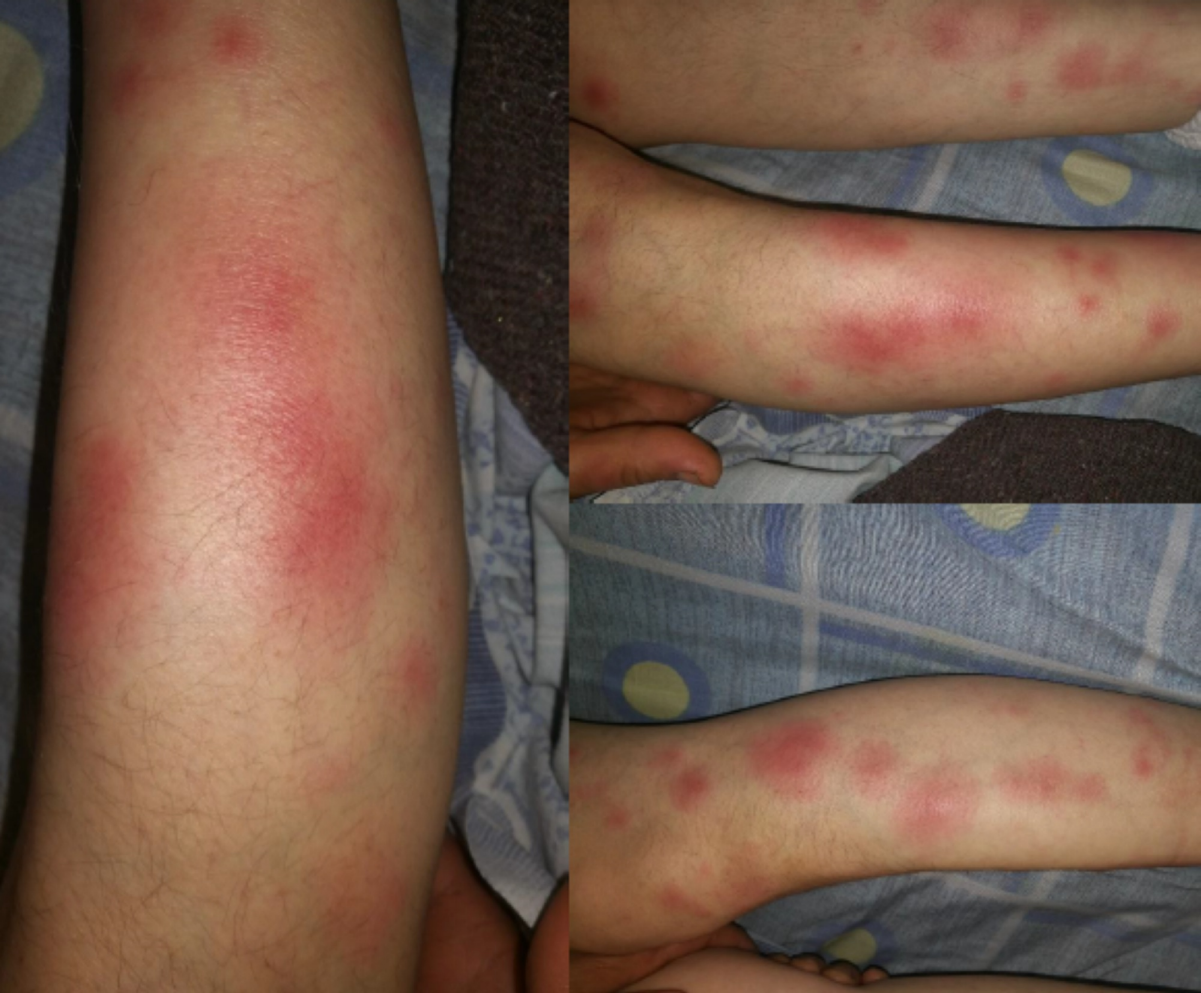
Erythema nodosum present on the bilateral legs

A 45-year-old woman, farmer by occupation, presented to our center with fever, swollen lymph nodes in the neck, and painful rash in the lower extremities. She was apparently well until before 15 days when she developed a fever and sore throat followed by a painful rash on her legs three days later. She visited a primary care center where she was prescribed a seven-day course of intramuscular ceftriaxone with a provisional diagnosis of streptococcal pharyngitis, which resolved the sore throat and fever but not the rash. When her fever relapsed several days later, this time accompanied by swelling of cervical nodes, the patient was referred to Nork Infectious Clinical Hospital in Yerevan for further evaluation and management. At presentation, her blood pressure was measured 115/70 mmHg, the temperature was 102˚F, pulse rate was 102 beats per minute, and the respiratory rate was 18 per minute. General examination revealed few enlarged tender cervical nodes on the left side of her neck and multiple red, firm, and tender lump-like rash (erythema nodosum) on both her legs (Figure 1).

Systemic examination was unremarkable. Blood workup revealed leukocytosis with relative lymphocytosis, raised erythrocyte sedimentation rate of 55 mm per hour, and C-reactive protein of 35 mg/L. We admitted her for a thorough evaluation. Serology for *Streptococcus*, Epstein-Barr virus, HIV, hepatitis B and C virus, *Cytomegalovirus*, *Brucella*, and leptospirosis were all negative. With unremarkable chest X ray and negative tuberculin test, we also eliminated sarcoidosis and tuberculosis as possible infectious causes of the erythema nodosum. She was diagnosed with tularemia based on latex agglutination testing, which came positive. A 14-day course of intramuscular streptomycin two grams daily divided into two doses was prescribed. Five days into treatment, the patient’s fever subsided, the buboes shrank, and the erythema nodosum resolved. The patient was discharged on the eighth day of admission with subsequent follow-up. Potentially exposed persons, particularly the family members, were advised to be vigilant about the symptoms, especially the development of fever for two weeks. They were informed about the water and food hygiene, which might have been the possible source of infection. The incidence was reported to the local health authority as a part of the standard protocol. The patient was doing better on the follow-up visit at three weeks with complete resolution of symptoms and return to a normal routine. 

## Discussion

*Francisella tularensis *is known to be very infectious, with as low as 10-25 organisms capable of causing disease, and hence considered a category A biowarfare agent. The incubation period is three to five days but can be as long as three weeks. The clinical features vary according to the route of transmission, inoculum size, and subspecies of the organism [2,5]. The most common symptoms are fever, sweating, headache, muscle pains, stiff joints, swollen lymph nodes, eye symptoms, ulcers, and breathing difficulty. Tularemia can present as ulceroglandular disease presenting with cutaneous ulcerations and marked lymphadenopathy, a glandular disease with marked lymphadenopathy, an oculoglandular form with preauricular lymphadenopathy, and conjunctivitis, an oropharyngeal disease with pharyngitis, stomatitis and cervical lymphadenopathy, gastrointestinal tract disease, respiratory disease, or as typhoidal tularemia. Complications include pneumonia, lung abscess, acute respiratory distress syndrome (ARDS), peritonitis, rhabdomyolysis, renal failure, and meningitis [2].

Ulceroglandular tularemia needs to be ruled out before diagnosing a tularemia case as oropharyngeal tularemia, which is not always easy. It is because ulceroglandular tularemia acquired by a tick or fly bites in the head and neck sometimes manifest neck lymph node enlargement only without a primary skin ulcer. An epidemiological evaluation can be helpful to hint at oropharyngeal tularemia. In our case, the history of sore throat suggested the oropharyngeal form [3]. Skin manifestations in the form of erythema nodosum and erythema multiforme can occur in tularemia as immune-mediated reactions [6]. However, it is unusual for oropharyngeal tularemia to manifest with erythema nodosum as a primary symptom, particularly one that persists throughout the illness. Although the cause of erythema nodosum is unknown in nearly half of cases, it is important to identify or exclude possible infectious causes of this condition, including tuberculosis, Valley fever, cat scratch disease, and, as illustrated in the case described herein, tularemia. 

The regional neck lymphadenopathy in the disease, along with fever and sore throat, can be easily misinterpreted as to be due to streptococci. In such a case, patients may receive penicillin, which does not kill the causative organism. In an outbreak of oropharyngeal tularemia in Turkey, delay in the diagnosis resulted in the development of suppurating lymph nodes in 40% of the patients [7]. In our case also, the patient was treated at the previous center for the streptococcal disease until she presented to our center. When the epidemiology is suggestive, tularemia should be considered in any case of fever of unknown origin [3]. As we did in our case, it is also important to exclude Epstein-Barr virus, HIV, hepatitis B and C virus, *Cytomegalovirus*, *Brucella*, and leptospirosis as the possible cause of the symptoms. The tuberculin test was negative; also, the tubercular disease is likely to have a slower course. Similarly, we also considered other conditions such as anthrax, *Pasteurella*, cat-scratch disease, and plague, but they were easy to exclude based on history, examination, the clinical course of the disease in our patient, and epidemiological evaluation. 

Although tularemia outbreaks are usually self-limiting, large outbreaks are possible if not taken care of. For instance, in 1999, the post-war situation in Kosovo (Serbia) was worsened by the socioeconomic crisis, making it difficult to prevent the tularemia outbreak [8]. As oropharyngeal tularemia is usually contracted via contaminated food and drinking water, surety of hygienic food and clean drinking water is a must to avoid an outbreak [3]. The occurrence of two outbreaks in the past (2003 and 2007) in Armenia indicates the presence of environmental factors that can lead to an epidemic [4]. Detected cases should also be reported to the concerned public health authority. Parenteral aminoglycosides, such as streptomycin or gentamycin, are the drugs of choice, whereas ciprofloxacin or doxycycline can be used in less severe case or mass casualty [3].

## Conclusions

Diagnosis and early treatment of tularemia can be difficult sometimes, especially in regions where it rarely occurs because the disease’s clinical manifestation mimics a wide variety of other infectious diseases. Strong clinical suspicion and appropriate diagnostic testing are imperative to identify and treat the illness. Although considered a rare disease, health care providers should be cautious about the possibility of the illness and remain vigilant about the various forms it can present with.
